# Primary care providers’ perceptions and experiences of family-centered care for older adults: a qualitative study of community-based diabetes management in China

**DOI:** 10.1186/s12877-021-02380-x

**Published:** 2021-07-23

**Authors:** Jiong Tu, Jing Liao

**Affiliations:** 1 School of Sociology and Anthropology, Sun Yat-sen University, Guangzhou, People’s Republic of China; 2 Global Health Institute, Institute of State Governance, Sun Yat-sen University, Guangzhou, People’s Republic of China; 3 Department of Medical Statistics & Epidemiology, School of Public Health, Sun Yat-sen University, No.135 Xingang West Road, Guangzhou, People’s Republic of China

**Keywords:** Family-centered care, Community-based primary care, Healthcare delivery, Carer involvement, Collaborative practices, Older adults, Qualitative study

## Abstract

**Background:**

Family-centered care, as a contemporary model of health service delivery, involves a mutually beneficial partnership between healthcare providers, patients and their families. Although evidence on the positive effects of family-centered care on older adults and their families is accumulating, less is known about the providers’ beliefs, attitudes and practices related to family-centeredness, especially regarding community-based primary healthcare services for the rapidly-ageing Chinese population.

**Methods:**

This study investigated Chinese primary care providers’ perceptions and experiences of family-centered care for older adults, using community-based diabetes management services as an example. Ten focus-group interviews involving 48 community health professionals were conducted. Major themes were identified using thematic analysis.

**Results:**

The interviews revealed that the providers acknowledged the importance of the family in older patients’ diabetes management, while their current scope of practice with the patients’ families was limited and informal. The barriers to implementing family-centered care were attributed to structural and environmental obstacles associated with the patients’ families and the community healthcare context and culture. To engage patients’ families more effectively, the providers suggested that family-centered values endorsed by their healthcare organizations and reinforced by policies, a trained interdisciplinary team of health professionals and community social workers, and also that the utilization of technology would be beneficial.

**Conclusions:**

Our study extends the evidence of family-centered care for older adults in Chinese community-based healthcare settings, contributing to the design of a tailored healthcare delivery model embodying ageing in place.

**Supplementary Information:**

The online version contains supplementary material available at 10.1186/s12877-021-02380-x.

## Introduction

With a rapidly ageing population and large proportion of older adults with chronic diseases and disabilities, the healthcare system in China has shifted its main objective from pure disease treatment to prevention and management at the population level [1]. The National Basic Public Health Service Program, established in 2009, targets the major chronic diseases (e.g. hypertension and diabetes), and particularly focuses on the health management of community-dwelling older adults [2]. Moreover, residents are able to register with a family doctor team, who can provide them with integrated preventive and primary care, plus continued referral services [3].

This community-based disease prevention and management approach offers the advantages of wide coverage and easy accessibility for patients and their families [4]. Family involvement in medical care occurs frequently, and has long been identified as a critical factor for health management [5]. Family members’ behavior concordance and daily support can compensate for the limited doctor-patient interactions, which are largely confined to a brief consultation time and care facilities [6]. The idea of actively engaging family in care conveys the vision of mutually beneficial partnerships between healthcare providers, patients and their families [7], and the family-centered care model has been promoted as a contemporary model of health service delivery over the last few decades [8].

While evidence of the positive effects of family-centered care on older adults and their families is increasing [9, 10], less is known about the providers’ beliefs, attitudes and current practices associated with family involvement in care delivery [11]. Some research, based in hospital or nursing home settings, suggests that providers generally agree about the benefits of involving patients’ families in planning and implementing care [12]. Contradictory to this belief, they tend to express negative attitudes about working effectively with families due to negotiation failures, power struggles, as well as a lack of time and incentives to educate patients’ families [13]. Providers question whether their scope of practice should include patients’ family members, considering the heavily burdened, under-staffed and task-oriented healthcare context [13, 14].

The discrepancies between the vision of family-centered care and its adoption by providers require further investigation, especially in the context of Chinese community-based primary healthcare. Research indicates that the traditional Chinese Confucian morals may cultivate harmonious doctor-family-patient relationships, whereby the family’s involvement in healthcare would happen naturally and be well-accepted by the providers [15]. It is also suspected that the community- and home-oriented practice environment of the community healthcare centers would better accommodate family-centeredness [16] compared with institutional healthcare settings [14, 17]. Therefore, the present study addressed these gaps in knowledge by investigating the Chinese primary care providers’ perceptions and experiences of family-centered care for older adults, as well as the barriers and facilitators that influence their partnerships with patients’ families, using the community-based diabetes management service as an example.

## Methods

### Setting and participants

We employed a qualitative analysis of group interviews with primary care providers. Focus group interviews [18] were carried out from March 2019 to July 2020 in Guangzhou, Guangdong, China. As the capital city of Guangdong, Guangzhou has a huge population base and 18% of which were aged 60 years and above, reaching 1.69 million by the end of 2018 [19]. Since the establishment of the National Basic Public Health Service Program in 2009, community healthcare centers have been responsible for health management for older adults, patients with hypertension and diabetes [20]. The diabetes management services include screening for high risk population, quarterly follow-ups for diagnosed patients, education and annual health checkups.

The interviewees were recruited from 26 community healthcare centers using a purposive sampling approach, to select a specific population being “focused” on a given topic [21]. These centers included both urban and rural areas, scattered across the 11 districts of Guangzhou. The participants were purposively selected to include all three roles public health practitioners, physicians and nurses, involving in community healthcare services provision. Potential participants were identified through the Guangzhou community healthcare centers registry, and were invited by phone and later met in person for interviews. For the purpose of this study, we specially recruited those involved in diabetes treatment and management.

### Data collection

In total, ten focus-group interviews involving 48 health professionals were conducted. The interviews, lasted approximately 90 min each, were carried out at either the interviewees’ respective health facilities or the Center for Disease Control and Prevention of Guangzhou when community health professionals from different areas gathered together for meetings. Each interview consisted of 4–6 health professionals, and was interviewed by two researchers (TJ and JL), including one moderator and one observer. Both researchers were well trained in qualitative data collection and analysis. The moderator was responsible for inquiry and situation control; while the observer took field notes to capture the contextual information during the interviews, such as which statement was made by which particular individual, as well as the non-verbal interactions and the group dynamic [21]. To obtain a broad range of information, interviews were semi-structured, and investigated health professionals’ daily work and practices related to diabetes management, the difficulties they encountered, and their attitudes and practices regarding family-centered care (see Additional file 1 for the interview guide). The interview guide was drafted based on our study questions and literature reviews, which was tested with one focus group and the revised version was later used in the formal interviews. Besides referring to the interview guide, the moderator also used probes to draw additional information by asking “Tell us more”, “Why you have that feeling?”, “Would you explain further?” Saturation was achieved after ten focus groups, indicating that the information provided by the health professionals began to be repetitive and no new themes were emerging [22].

### Data analysis

All of the group interviews were audio-recorded and transcribed verbatim in Chinese via iFLYTEK transcription software. The two authors then further refined these transcripts and coded them with Nvivo 11 using thematic analysis [23]. We followed a positivist approach in thematic analysis, adopting a “coding reliability” thematic analysis that concerns about objective and unbiased coding [24]. According to this thematic analysis approach, we used a codebook for the analytic process. The codebook was informed by the research questions and focused particularly on the health professionals’ views and practices (barriers and facilitators) regarding family-centered care. Two researchers (TJ and JL) analyzed the data independently and then compared with each other. They read and re-read the whole transcripts, in the process deductively identified contents relevant to the predetermined topics of the codebook, and then summarized the main points expressed in these topics into themes. Basically there is not much difference in the themes identified by the two coders, except the ways for expression. The two researchers then discussed and confirmed the themes and subthemes. The findings were presented in the manuscript, and verbatim quotations were chosen and translated into English by the authors to provide support for each theme.

### Rigour

The rigour of this study was ensured by careful applications of the study transparency, credibility, dependability, comparativeness and reflexibility [25]. Specifically, transparency was achieved by clearly describing the research procedures that all followed relevant guidelines. Credibility was ensured through maintaining the detailed records and presentation of the data. We not only recorded and transcribed the interviews, but also recorded the interview settings and the non-verbal communications among the interviewees to complement the verbal transcript. Dependability was ensured by the two authors coding the data independently to avoid bias. Comparability was enhanced by situating and comparing our study with the broader literature. Regarding reflexibility, although we did not use “reflexive” thematic analysis as mainly endorsed by Braun and Clarke [24], we were aware of that there were different approaches within thematic analysis, and we chose “coding reliability” thematic analysis for the current analysis given it better suited our study purposes.

The study was approved by the School of Public Health, Sun Yat-sen University Institutional Review Board (Approval no. 2019–064). At the time of conducting the interview, the method and aim of the qualitative study were explained to participants. All the participants provided written informed consent. Participants’ personal information was collected and stored in a separate file. Individual name was anonymized, replacing by an allocated ID number (e.g. Interviewee1) in the transcript.

### Findings

#### Interviewee characteristics

As shown in Table 1, 67% of our interviewees were female, the majoirty were aged between 30 and 39 years. Nearly half of the interviewees were physicians, over one third were nurses, and the rest were public health practitioners. Having practiced in the community for an average of 11.6 years (SD 8.4), our interviewees’ rich experiences greatly contributed to our study.

**Table 1:** Characteristics of the primary care providers interviewed (*n* = 48)

Characteristics	N (%)
**Age, year**	
20–29	7 (14.9)
30–39	25 (52.1)
40–49	14 (29.0)
50–60	2 (4.0)
**Gender**	
Male	12 (33.3)
Female	36 (66.7)
**Profession**	
Physician	20 (41.7)
Nurse	19 (39.6)
Public health practitioner	9 (18.7)
**Years in practice**: mean year (SD^†^)	11.6 (8.4)
**Community type**	
Rural	16 (33.3)
Urban	32 (66.7)

^†^SD: standard deviation

### Attitudes to and experiences of family-centered diabetes care

#### Acknowledging the importance of family involvement

Nearly all of the interviewees acknowledged the importance of family involvement. They recognized that family arrangements fundamentally impacted patients’ management behaviors, and the family’s coordination was essential for successful diabetes management, particularly for older males who relied on their wife for their daily management (e.g. meal preparation) and became forgetful with age.

Family monitoring is effective [for older adults], say, males like me do not know much about cooking. Many elderly [males] do not cook at all. No matter how much you educate him, he still relies on his wife [for meal preparation], so coordinating the whole family to change will be better. (Interviewee18, physician, male)

Some older adults are stubborn and forgetful. If family members are around to help and monitor, it’d be much better. (Interviewee8, physician, female)

The providers further noted that, for health professionals, the patients’ family members may greatly shift the diabetes management work and sustain the health education efforts beyond the clinical settings, if a shared understanding can be achieved. On the other hand, for the family members, the providers’ professional knowledge could help them to supervise older adults with diabetes to make behavior changes.

The family member is familiar with the patient’s living environment, can interact with the patient anytime, and is familiar with the patient but we [health professionals] can hardly go into the family. We can only update his/her [the patient’s] status when he/she comes [for an outpatient visit], or make a telephone call to intervene; we can only do these things. Family involvement is indeed good. (Interviewee20, head nurse, female)

Sometimes a couple came to visit, and I could feel that one partner did not follow the health guide, so the other [the wife] brought the husband to the doctor. ‘Listen, the doctor said that!’ she’d say. I knew he did not listen to the wife but, if the doctor suggested, he’d listen, so the two came together. The wife persuaded the husband through our doctor’s mouth. (Interviewee30, physician, female)

#### Limited scope of practice with the family

Albeit appreciating the importance of family involvement, the providers indicated that their current scope of diabetes management was primarily patient-oriented, and that the families were only involved informally. The providers were open to the family’s presence during the consultation, allowed the family members to obtain medication for the patient, and would provide them with brief advice about the patient if the consultation time allowed. They also invited the patients’ families to attend education programs and health promotion lectures on diabetes, even though only a handful of family members would attend.

In most cases, it is one family member who helps the other to take medicine. Probably the patient has a mobility issue or has to work. We may also ask [the family member] a little about the patient’s blood sugar or blood pressure levels. (Interviewee31, physician, female)

They commented that, although the family doctor program had been formally implemented in Guangzhou since 2018, progress was slow and considerable difficulties with it had arisen. According to their perception, the family doctor program so far had been focused on individual residents only, rather than caring for the family as a whole, and many services were not yet in place.

The idea [to involve the family] has been there for some time as, when we promoted the family doctor program, it included the idea of family-centeredness. However, it has been implemented slowly. At the beginning, we planned to get a whole family to register with one [community] doctor. However, it ended up with individual patients registering with individual doctors. (Interviewee23, public health practitioner, female)

### Barriers to involving the family in diabetes care

The providers perceived several barriers as affecting their practice of family-centered care, as listed in the upper panel of Table 2 and detailed below.

**Table 2:** Factors influencing Chinese primary care providers’ practice of family-centered care

Barrier	Explanation
**Community healthcare context**	Shortage of staff
	Heavy workload
**Institutional culture**	A focus on disease treatment and control
	Task-performance oriented
**Family structure and arrangement**	Patient’s family dynamics
	Small family living far-apart
	Weakened family connections
**Facilitator**	**Explanation**
**Institutional endorsement**	Clear guidance and support
	Reinforcement of family-centered policies
	Trained interdisciplinary teams
**Community partner collaboration**	Collaboration with social service organizations
	Promotions by the government
**Technology utilization**	Flexible and timely communications with the patient family
	Mobilizing intergenerational support

#### Shortage of staff and heavy workload

The shortage of staff and heavy workload in the community healthcare center made actively involving the patients’ families challenging. The providers mentioned that there were increasing numbers of adults with chronic conditions like diabetes in their catchment area, and many articulated a high imbalance in the provider-patient ratio, such as “*There were over 1000 adults with diabetes [in my community], while we only have one [public health practitioner] in charge of [all] diabetes cases”.* They complained about the difficulties of delivering the required diabetes management services with the limited personnel, and commented “*the so-called four follow-up clinical visits [per year] is merely a formality”.* Besides, they were overwhelmed with the other services of the National Basic Public Health Program, and were also the frontline response to public health emergencies.

During the COVID-19 pandemic, although the pandemic was not very serious here, we were on alert. Besides our regular services, we need to track each patient’s travelling path over the past 10 days, and conduct a door-to-door survey to check the residents’ health conditions. Recently, we took charge of a physical checkup for those travelling to China from abroad, and are about to carry out a physical checkup in primary school. (Interviewee23, nurse, female)

Thus, many interviewees indicated they could hardly spare any time or energy to engage the patients, such as organizing patient education groups, let alone engage with the patients’ families, which was not within their practice scope. After all, they may not be rewarded for devoting extra efforts to something outside their work duties, whilst at the same time running the risk of being alienated.

Family-centeredness is very good, but we could hardly go that far. It’s best if we can convince the [patient’s] family … but we don’t have the energy to do that, and we’re under constant pressure for various tasks. [In my community healthcare center], we don’t even have a person fully in charge of diabetic education and management. (Interviewee34, physician, female)

#### A disease- and task-performance-oriented healthcare culture

The providers suggested that other systemic barriers within their practice environment also prevent them from engaging with the patients’ families. They commented that their main focus with diabetes management was to control the disease symptoms from a bio-medical perspective. Despite appreciating the idea of taking a holistic view of the person with diabetes, including the family, it was very hard for them actually to change their familiar method of practice.

The providers’ practice behavior was directly related to the work culture of the community healthcare center. Their work performance was mainly evaluated based on figures like the percentage of diabetes cases under control over all registered residents with diabetes. They thus paid more attention to achieving quantity rather than improving quality, and were occupied with paperwork and administration duties. They felt uncertain that taking the initiative to involve the family in the practice would be appreciated by their colleagues, managers and the organization. Without clear support and incentives from the administration, they were also worried about creating negative consequences and work conflict.

Our evaluation for [diabetes] management is based on the rate of diabetes under control over all registered residents with diabetes. Sometimes, we have to find more [potential patients] to meet the quota. We devote all of our efforts to achieving the figure. (Interviewee34, physician, female)

The leader required us to prepare a perfect file and record for evaluation. This happened twice a year, and each evaluation lasted for 1-2 months. If the evaluation result was poor, the [government] fund to our center would be cut. We had to devote more time to that [paper work], and so the amount of time left to spend with the patient and their family was reduced. If there had been fewer evaluation indices, we could have offered more practical services to the patients. (Interviewee33, nurse, female)

#### Family dynamics and changing structures

Although acknowledging that traditional Chinese family culture may be a valuable resource for incorporating patients’ families into healthcare, the providers indicated that the family members’ involvement in older adults’ diabetes management would largely depend on their willingness to do so, which varied according to the family dynamics. Some exemplified the practical difficulties preventing family members’ involvement, such as the absence of significant others (e.g. being widowed), the family members’ own poor health condition, and other family and work obligations.

When an old woman has diabetes, it is ideal if her husband and daughter care for her and take part in the disease management but, often, the reality is that her husband doesn’t pay much attention to it and her daughter is too busy to get involved. Besides, many families would not tell us [health professionals] much about their family issues. This involves their family relationships, which are difficult for us to intervene in. (Interviewee36, physician, female)

Difficulties were also attributed to the changing family structure and contemporary living arrangements. The traditional way of family care, within the same household, has been fundamentally challenged by the dramatically decreased family size and increase in the number of elderly ‘empty nesters,’ associated with the former one-child policy and urbanization. Providers from rural areas commented that the younger generation has moved to the cities, so only older adults are left behind, with insufficient family support. Their comments were echoed by providers practicing in urban healthcare centers, indicating weakened family connections.

Sometimes, the situation does not permit the younger generation to be involved. In our town, there are many hollow villages, with only older adults living there. Young people move to the city to work or study, and only return during vacations. How can you expect them to take care of their parents? (Interviewee35, physician, male)

In our [urban] community, older adults often live independently of their [adult] children. Some are close to their children’s houses while others are far apart but, in general, young people are busy with their own business, and rarely accompany their parents to our center. (Interviewee38, nurse, female)

### Facilitators for family-centered care in community

In view of the barriers outlined above, our interviewees nonetheless provided constructive suggestions about how to facilitate the attempts to improve family-centered care in community healthcare centers (Table 2 lower panel).

#### Endorsing a family-centered practicing environment

Overloaded by routine primary care services, the providers stated that it would be beneficial to have clear guidance and support from the administrators and leaders of their institutions on how they might enhance the involvement of the family in patients’ care. They reiterated that incentives and resources were needed to overcome the current obstacles.

The community healthcare center leader’s support is essential, because family involvement programs for diabetic patients are not our routine work and do not count toward our performance-based assessment. Only with the leader’s support will we have the staff and resources to carry it out. (Interviewee7, nurse, female)

Many resources and a lot of energy are wasted on administrative work … If family-centeredness was included as one of the evaluation indices, replacing an existing useless one, probably we would have more motivation to carry it out. (Interviewee35, physician, male)

The further reinforcement of family-centered policies, such as the family doctor program, were essential to bring about changes in their current practicing environment. Some suggested that reform on the providers’ side, such as the rearrangement of the healthcare team, would be helpful.

We now organize our healthcare providers into teams [as suggested in the family doctor program]: a physician, a public health practitioner and a nurse. They can then share the healthcare work: ideally, the physician focuses on the medical consultation, the public health practitioner conducts the follow-up, while the nurse gathers both sets of information and spends more time communicating with the older adults and their families. (Interviewee23, nurse, female)

#### Collaborating with community partners

Moreover, the providers indicated that the coordination between community healthcare centers, neighborhood committees (or village committees in the rural areas) and social service organizations should be strengthened in order to facilitate family involvement. They recognized that these community partners had wider, closer connections with the residents and their families, and could ideally compensate for the health professionals’ limited skill set.

Community healthcare centers can only access those who seek medical care. The neighborhood committee and the family service center [a type of social work organization] can more easily access the residents and their families. However, our center collaborates very little with these community-based organizations. The other two institutions will not do things that are currently not their responsibility. (Interviewee1, nurse, female)

Our target population and services overlap those of the neighborhood committee and the social service organization. They do not have medical expertise but we do, and they are more familiar with the local residents than us; hence we can help each other, but we do not collaborate with them much. A consensus has not been reached. (Interviewee42, nurse, female)

As acknowledged by the providers, their current chronic disease management services rarely involved community partners. However, this collaboration could work smoothly and successfully if it were promoted by the government, as in the case of combatting the COVID-19 pandemic.

During the COVID-19 outbreak, all community parties were mobilized and united: the neighborhood committee tracked and identified any positive case, notified us to make further medical verification, and then we managed the patient and their family together. If chronic illness management can be organized like an anti-pandemic effort, it would work far better. (Interviewee33, nurse, female)

#### Advancing connections with patients’ families through the use of technology

The providers suggested that mobile technology and telecommunication applications would facilitate the communication and connections with the patients and their families. Some of them had experience of organizing online patient consultations and education groups, especially during the peak of COVID-19, when the regular onsite medical services were interrupted.

The providers at our community center have organized remote consultations and live-streamed health education via popular social media and applications, like WeChat. Their record was to have over a hundred local residents participate online, a much larger group than we could normally achieve in offline education courses, which usually consist of, say, around 20 people. (Interviewee23, nurse, female)

They commented that these technology-powered initiatives provided more flexibility and possibility to collaborate with the patients’ families, and were particularly welcomed by the younger generation, who can assist older adults to adopt new technologies and behavior change.

We set up two [WeChat online] groups: one for diabetic patients and one for those with hypertension. We share health information [in the group] regularly … I believe this is the trend. For these older adults, who are unfamiliar with this new method of information communication, I encourage their children to teach them slowly. (Interviewee40, physician, female)

We need to take advantage of the family’s influence. The information about diabetes or high blood pressures is more likely to reach young people. They could help us to spread the health information to their family, and promote behavior change within the family. (Interviewee45, public health practitioner, male)

## Discussion

The present study explored Chinese primary care providers’ perceptions and experiences of family-centered diabetic care for community-dwelling older adults. Our findings revealed that the providers appreciated the importance of the family’s involvement in patients’ diabetes management, while their current scope of practice with the family was limited and informal. Barriers and facilitators related to implementing family-centered care for older adults in community healthcare settings were identified.

Our study contributes to the family-centered care literature by generating empirical evidence about the community-based primary healthcare setting in a Chinese culture context. Different from previous studies that mainly applied the principles of family-centered care in the context of hospitals [26, 27], long-term care [28], and end-of-life and palliative care [13], our study directly examined its application to community-based primary care services. As revealed by our interviews, the interactions between healthcare providers, the patients and their families were rather frequent, dynamic and complex at the community health care level. This finding is in line with prior evidence that the providers are typically regarded as the authority figures in a hospital setting; whereas the family members tend to exert a greater influence on the patients’ care plan in a home- and community-based care setting [29]. Moreover, our observations of the family’s heavy involvement in patient management are also in agreement with prior studies on patient care activities [30] and medical decision-making [15], which are particularly apparent against the Chinese family-centered Confucian culture associated with a strong moral obligation among family members [31]. Regarding diabetes management, that permeates patients’ daily routines, our previous investigation has also shown that the Chinese family is un/consciously involved in managing patients’ health and behaviors [32]. As an invaluable source of informal care, the family may nevertheless impede diabetes management due to their misunderstanding and poor health literacy. The providers interviewed in our present study were well aware of the substantial influence of the Chinese family on older adults’ diabetes management, and interacted with the patients’ families in their practice to varying degrees. When a shared understanding could be reached between them and the patient’s family, the providers also acknowledged a mutual beneficial partnership, as highlighted in the family-centered care model [7].

Yet, our study further revealed that the providers’ practice of family-centered care was limited and informal. Although the family doctor program had been conducted for several years, their current practice was primarily focused on individual patients only and from a bio-medical perspective. The barriers mentioned include a shortage of staff and heavy workload, a task-performance-oriented healthcare culture, and the family dynamics and changing structures. Similar obstacles have been reported in previous studies on institutional [13, 33] and home-based [14, 29] healthcare services for older adults, where professionals found coordinating or supporting the needs of the patients’ families required extra effort and time, lay outside their job responsibilities, and might not be supported by the current healthcare culture.

The similarity between the barriers that were identified conflicted with our hypothesis that a community-based care setting would accommodate family-centeredness better than institutional healthcare. It is noted that our interviewees tend to attribute their low involvement with the patients’ families to structural and environmental barriers within both the patients’ families and the healthcare system. This may actually reflect attitudinal issues and suggests that the primary care providers may feel more comfortable and confident about practicing a paternalistic care model [8] rather than partnering with patients and their families, regardless of their awareness of the importance of family-centeredness. Apart from the providers’ attitudes, contextual factors also appear to be key determinants in influencing their actual involvement with the patients’ families. Limited resources and a healthcare culture that lacks a clear vision for family-centered care have been found to be the strongest factors affecting doctor-patient-family interactions within clinical practice [34]. The majority of community healthcare centers in China still struggle due to a shortage and poor competency of health workers [35]. Without sufficient resources, welfare benefits, concrete implementation strategies and supporting policies devoted to family-centered care by the centers plus health administrations at local or municipal levels, providers would still spend most of their time on disease treatment and control, attending to cost and efficiency as primarily required by the evaluation matrices, rather than truly meeting the care needs of the patients and their families [36].

To convert family-centered care from theory to practice, the providers interviewed emphasized that family-centered values should be endorsed by their healthcare organizations and reinforced by policies. Only when supported by their colleagues and appreciated by the administrators and the organization will providers feel encouraged to make proactive attempts to engage with the patients’ families. Additionally, they indicated that inter-professional collaboration between healthcare providers and community social partners would be beneficial, as this would enable both parties to share the communication burden and provide social- and medical- supports to the older residents’ families. As highlighted by Ruggiano and Edvardsson [37], social workers could help to overcome the cultural and structural barriers to extending person-centeredness into family-oriented long-term care, through connecting the fragmented medical and non-medical services, empowering older patients to participate actively in their care plans, and lobbying policy-makers and administrators about older adults’ needs. Furthermore, the providers suggested that utilizing telecommunication technology and social media would significantly increase the feasibility and flexibility of communicating with patients and their families who may live far apart, and particularly suit the preference of the younger generation. These reflections are well supported by the literature, where it has been found that the use of technology strengthens intergenerational communication [38], and ensures that medical prescriptions and advice are timely and accurately conveyed between healthcare providers, social workers, and patients’ families [29], which tends to be particularly valuable during public health crises [39].

### Strengths and limitations

We interviewed a diverse group of public health practitioners, physicians and nurses from all 11 districts of Guangzhou across both rural and urban areas, covering a broad range of perspectives. Frontline primary care providers spoke of their observations and experience of the family’s involvement in routine practices for older adults before, during and after the COVID-19 pandemic. These findings thus provide unique evidence for healthcare providers, researchers and policy-makers who are interested in planning and implementing family-centered care in the community. Nevertheless, we note that our participants were recruited in a first-tier city in China, and the extent to which findings here may be applicable to less developed settings needs further investigations. Individuals, such as administrators and senior leaders, who might offer different perspectives on family-centered care, were not included. More explorations on the potential meaningful differences between specific provider groups and between locations are required.

## Conclusions

In summary, our qualitative study examined Chinese primary care providers’ perceptions and practices of family-centered care for older adults with diabetes in the community, providing cultural- and contextual-specific evidence regarding family-centeredness in health services. The healthcare providers interviewed generally perceived the importance of the family’s involvement in older patients’ diabetes management. Their limited engagement with the family was related to the family dynamics and an overloaded task-performance-based healthcare culture, while a family-centered practice environment endorsed by the organizations and policies, wide collaborations with community partners and the utilization of technology, would facilitate the implementation of family-centered care. The understanding of the dynamics between the providers, patients and their families in community healthcare settings would allow a clearer distinction to be drawn between the providers and patients’ families’ roles and needs regarding healthcare planning for China’s rising ageing population.

## Supplementary Information

**Additional file 1.** Interview guide for community healthcare providers.

## Data Availability

The audio records and the transcripts collected during the current study are stored in a file on a local computer of Department of Public Health, Sun Yat-sen University for data security, which are available from the corresponding author on reasonable request. Participants’ personal information is confidential and is not shareable.
