# Supplementary material for: Primary care providers’ perceptions and experiences of family-centered care for older adults: a qualitative study of community-based diabetes management in China
Source: BMC Geriatr. 2021 Jul 23;21:438. doi: 10.1186/s12877-021-02380-x (PMC8299165; doi:10.1186/s12877-021-02380-x)
Supplement: Supplementary file 1 — Additional file 1. Interview guide for community healthcare providers. [file 12877_2021_2380_MOESM1_ESM.docx]

**Additional file 1. Interview guide for community healthcare providers**

1. What do you and your institution provide in diabetes management for older adults?
2. What affect older adults’ self-management? Is there any family related factor?
3. Have you and your institution involved the patients’ families in diabetes management? What have you done?
4. To what degree the families are involved? What’s the effect?
5. Do you have any opinion or thoughts about family-centered care? How should it be designed?
6. What do you think about the Chinese family culture? How will it influence the implementation of family-centered care?
7. What are the challenges to implement family-centered care in community chronic illness management (diabetes care in particular)?
8. Any suggestion on how to better involve the family? What should be done within your institution? What support are needed beyond your institution?

**Basic information for participants in group interviews**

Age, gender, profession, years of practice, affiliated institution
